# Purinergic signaling via P2X receptors and mechanisms of unregulated ATP release in the outer retina and age-related macular degeneration

**DOI:** 10.3389/fnins.2023.1216489

**Published:** 2023-07-11

**Authors:** Haydn Molcak, Kailun Jiang, Christopher J. Campbell, Joanne A. Matsubara

**Affiliations:** ^1^Matsubara Lab, Faculty of Medicine, Department of Ophthalmology and Visual Sciences, Eye Care Centre, Vancouver, BC, Canada; ^2^Paragon Ventures Inc, Vancouver, BC, Canada

**Keywords:** purinergic signaling, age-related macular degeneration, ATP, complement, inflammasome, P2X

## Abstract

Age-related macular degeneration (AMD) is a chronic and progressive inflammatory disease of the retina characterized by photoceptor loss and significant central visual impairment due to either choroidal neovascularization or geographic atrophy. The pathophysiology of AMD is complex and multifactorial, driven by a combination of modifiable and non-modifiable risk factors, molecular mechanisms, and cellular processes that contribute to overall disease onset, severity, and progression. Unfortunately, due to the structural, cellular, and pathophysiologic complexity, therapeutic discovery is challenging. While purinergic signaling has been investigated for its role in the development and treatment of ocular pathologies including AMD, the potential crosstalk between known contributors to AMD, such as the complement cascade and inflammasome activation, and other biological systems, such as purinergic signaling, have not been fully characterized. In this review, we explore the interactions between purinergic signaling, ATP release, and known contributors to AMD pathogenesis including complement dysregulation and inflammasome activation. We begin by identifying what is known about purinergic receptors in cell populations of the outer retina and potential sources of extracellular ATP required to trigger purinergic receptor activation. Next, we examine evidence in the literature that the purinergic system accelerates AMD pathogenesis leading to apoptotic and pyroptotic cell death in retinal cells. To fully understand the potential role that purinergic signaling plays in AMD, more research is needed surrounding the expression, distribution, functions, and interactions of purinergic receptors within cells of the outer retina as well as potential crosstalk with other systems. By determining how these processes are affected in the context of purinergic signaling, it will improve our understanding of the mechanisms that drive AMD pathogenesis which is critical in developing treatment strategies that prevent or slow progression of the disease.

## Introduction

Purinergic signaling is a form of extracellular signaling involving purine and pyrimidine nucleotides and nucleosides that act on purinergic receptors to mediate numerous cellular functions (Burnstock, 2008). In total, there are three distinct classes of purinergic receptors known as P1, P2Y, and P2X receptors. P1 receptors are G protein-coupled receptors (GPCRs) that respond to adenosine (Burnstock, 2018). P2Y receptors are GPCRs that respond to nucleotides such as adenosine triphosphate (ATP), adenosine diphosphate (ADP), adenosine monophosphate (AMP), adenosine, uridine triphosphate (UTP), uridine diphosphate (UDP), and UDP-glucose (Burnstock, 2018). P2X receptors are unique and function as ligand-gated ion channels that respond exclusively to extracellular ATP (eATP) (Burnstock, 2018). Purinergic signaling represents a set of phylogenetically ancient pathways that play a critical role in numerous cellular processes, bodily systems, and developmental stages, including proliferation, differentiation, migration, apoptosis, embryogenesis, organogenesis, and aging (Burnstock and Dale, 2015; Huang et al., 2021). Within the eye, purinergic signaling has been investigated for its role in the development and treatment of ocular pathologies such as age-related macular degeneration (AMD), glaucoma, and diabetic retinopathy (Ye et al., 2021).

AMD is a leading cause of visual impairment and accounts for approximately 9% of all cases of blindness worldwide (Wong et al., 2014). The pathophysiology of AMD is complex and multifactorial, driven by a combination of non-modifiable risk factors (e.g., aging, genetic predisposition) and modifiable risk factors (e.g., smoking, hypertension, body mass index, hypercholesterolemia, nutritional intake, UV light) that contribute to overall disease onset, severity, and progression (Ambati and Fowler, 2012; Wong et al., 2014; Mitchell et al., 2018; de Jong et al., 2020; Stahl, 2020). AMD can be subdivided into early, intermediate, and advanced stages, with the advanced forms characterized by photoreceptor loss in the macula, the region of the retina responsible for central vision. There are two forms of advanced AMD. Wet (or exudative) AMD (~10% of cases) develops due to choroidal neovascularization (CNV), a form of abnormal angiogenesis in the choriocapillaris layers of the choroid, which results in the growth of neo-vessels breaching Bruch’s membrane, causing photoreceptor death (Mitchell et al., 2018). Dry (or non-exudative) AMD (~90% of cases) is a slowly progressing degenerative process whereby regions of retinal pigment epithelium (RPE), a monolayer of cells that combine with Bruch’s membrane and form the outer blood-eye barrier, undergo cell death leading to geographic atrophy (GA) (Mitchell et al., 2018). The early stages of both forms are characterized by the buildup of drusen deposits, which are composed of glycoproteins, lipids, and immunogenic factors. The drusen accumulate in the extracellular space between the RPE and Bruch’s membrane and provoke a chronic proinflammatory milieu that triggers the development of AMD (Mitchell et al., 2018).

Despite the emergence of anti-vascular endothelial growth factor (VEGF) agents to treat wet AMD, there are currently no therapies available to prevent the development of dry AMD aside from nutritional supplementation (AREDS formulation) (Age-Related Eye Disease Study Research Group, 2001). However, in 2023, the FDA approved pegcetacoplan, a complement C3 inhibitor, to slow the progression of GA, providing some promise for those with late-stage GA. It is also possible for a portion of patients with wet AMD to develop severe vision loss and blindness by developing GA over time, further emphasizing the complex nature of AMD pathogenesis (Holz et al., 2014; Rasmussen and Sander, 2014; Agarwal et al., 2015; Chen and Kaiser, 2020). Thus, understanding the multiple mechanisms that drive AMD and the interplay between wet and dry forms, is crucial in developing treatment strategies that not only slow the progression of the disease, but ultimately prevent its development.

In the present review, we explore purinergic signaling and mechanisms of unregulated ATP release in the outer retina, and its potential significance in AMD pathogenesis. While there is evidence for an interplay between purinergic signaling and the mechanisms associated with AMD pathogenesis such as complement dysregulation, inflammasome activation, and sublytic membrane attack complex (MAC) deposition, few studies have addressed their detailed interactions. We first begin by identifying what is known about purinergic receptors in cell populations of the outer retina along with potential sources of eATP required to trigger purinergic receptor activation. Next, we examine evidence in the literature that the purinergic system accelerates AMD pathogenesis leading to apoptotic and pyroptotic cell death in RPE, photoreceptors, and choroidal cells.

## Purinergic signaling

The purinergic system is a form of cell signaling in which both purine and pyrimidine nucleotides and nucleosides act on extracellular purinergic receptors (Burnstock, 2008). As outlined above, purinergic receptors are divided into two classes known as P1 and P2 receptors. P1 receptors are GPCRs that respond to the nucleoside adenosine (Burnstock, 2018). In contrast, P2 receptors are nucleotide receptors that are further subdivided into P2X and P2Y receptors. P2Y receptors are GPCRs that respond to several nucleotides such as ATP, ADP, AMP, UTP, UDP, and UDP-glucose (Burnstock, 2018). P2X receptors are ligand-gated ion channels that respond exclusively to eATP (Burnstock, 2018). This review will focus on P2X receptors which are composed of two transmembrane domains with cytoplasmic amino-and carboxyl-terminals that polymerize to form homotrimeric or heterotrimeric channels permeable to cations such as sodium, potassium, and calcium (Jacobson et al., 2020). In total, seven homotrimeric P2X receptors (P2X1-7) and several heterotrimeric P2X channels with hybrid properties exist (Ralevic and Burnstock, 1998; Jacobson et al., 2020; Santiago et al., 2020; Illes et al., 2021). Table 1 outlines the unique features of P2X1-7 receptors based on their functional and pharmacological properties (Illes et al., 2021). For a comprehensive review of purinergic signaling and current developments in this field, please see Burnstock (2006, 2017).

**Table 1:** P2X receptors showing their molecular, pharmacologic, and functional properties [Adapted from Illes et al. (2021)].

	P2X1	P2X2	P2X3	P2X4	P2X5	P2X6	P2X7
ATP EC50 (μM)	0.56–0.7	2–8	0.5–1	1–10	0.44–10	12	100
Desensitization	Rapid (<1 s)	Slow (>20s) or no desensitization	Rapid (<1 s)	Slow (>20s)	Slow (>20s)	Slow (>20s)	Slow (>20s)
Function	Non-selective cationic channel	Non-selective cationic channel	Non-selective cationic channel	Non-selective cationic channel Permeability for Ca2+ among highest in P2X family	Non-selective cationic channel Permeable to chloride ions	Non-selective cationic channel	Non-selective cationic channel
Large Pore	No	Yes	No	Yes	-	-	Yes
Functional Heterotrimers	P2X2, P2X4, P2X5	P2X1, P2X3, P2X5, P2X6	P2X2	P2X1, P2X6	P2X1, P2X2	P2X2, P2X4	P2X4
Location/ Cellular Expression Within the Retina	Inner plexiform layer, Muller cells, endothelial cells, glial cells	Amacrine cells, Muller cells, neurons	Amacrine cells, neurons	Glial cells, endothelial cells, neurons, horizontal cells of retina, amacrine and ganglion cells of the retina, Muller cells	Amacrine cells	Nerve fiber layer	Plexiform layers, horizontal cells, photoreceptors, amacrine cells, ganglion cells, glial cells, RPE, choroid, Muller cells

Due to its well-established role in several inflammatory processes, a significant quantity of research has been performed surrounding P2X7. However, normal extracellular concentrations of ATP are approximately 10 nM under steady-state conditions, and the half maximal effective concentration (EC50) for P2X7 receptor activation is approximately 100 μM. Furthermore, once ATP is released, it can be rapidly degraded by ecto-enzymes yielding ADP, AMP, and adenosine, further decreasing the concentrations of ATP available for P2X activation. This means that the concentration of ATP required to activate P2X7 receptors is considerably higher than that found under physiological conditions leading to past debate surrounding its physiological relevance, especially in early stages of disease or inflammatory processes (Yang et al., 2011). However, with the novel introduction of plasma membrane luciferase (pmeLUC) which has made direct measurements of eATP possible, the concentration of eATP has been shown to reach 100–200 μM (Pellegatti et al., 2005, 2008; Morciano et al., 2017; Romagnani et al., 2020). Additionally, positive allosteric modulators acting at P2X7 are released into circulation during inflammation, thus further increasing the affinity of P2X7 for ATP (Tomasinsig et al., 2008; Kahlenberg and Kaplan, 2013; Di Virgilio et al., 2018). Secreted or membrane-bound ecto-kinases such as adenylate kinase, nucleoside monophosphokinases, and nucleoside diphosphokinases can also phosphorylate nucleosides to produce AMP, ADP, and ATP (Schwiebert and Zsembery, 2003). Taken together, there are several mechanisms whereby the concentration of eATP is capable of activating any and all of the P2X family of receptors. While questions remain surrounding the underlying pathways of P2X7 signaling in AMD, there is a significant gap in the literature surrounding other members of the P2X family of receptors, including novel and hybrid properties of heterotrimers, that may play an important role in various cellular and inflammatory processes.

## Purinergic signaling in the retina

**Figure 1:**
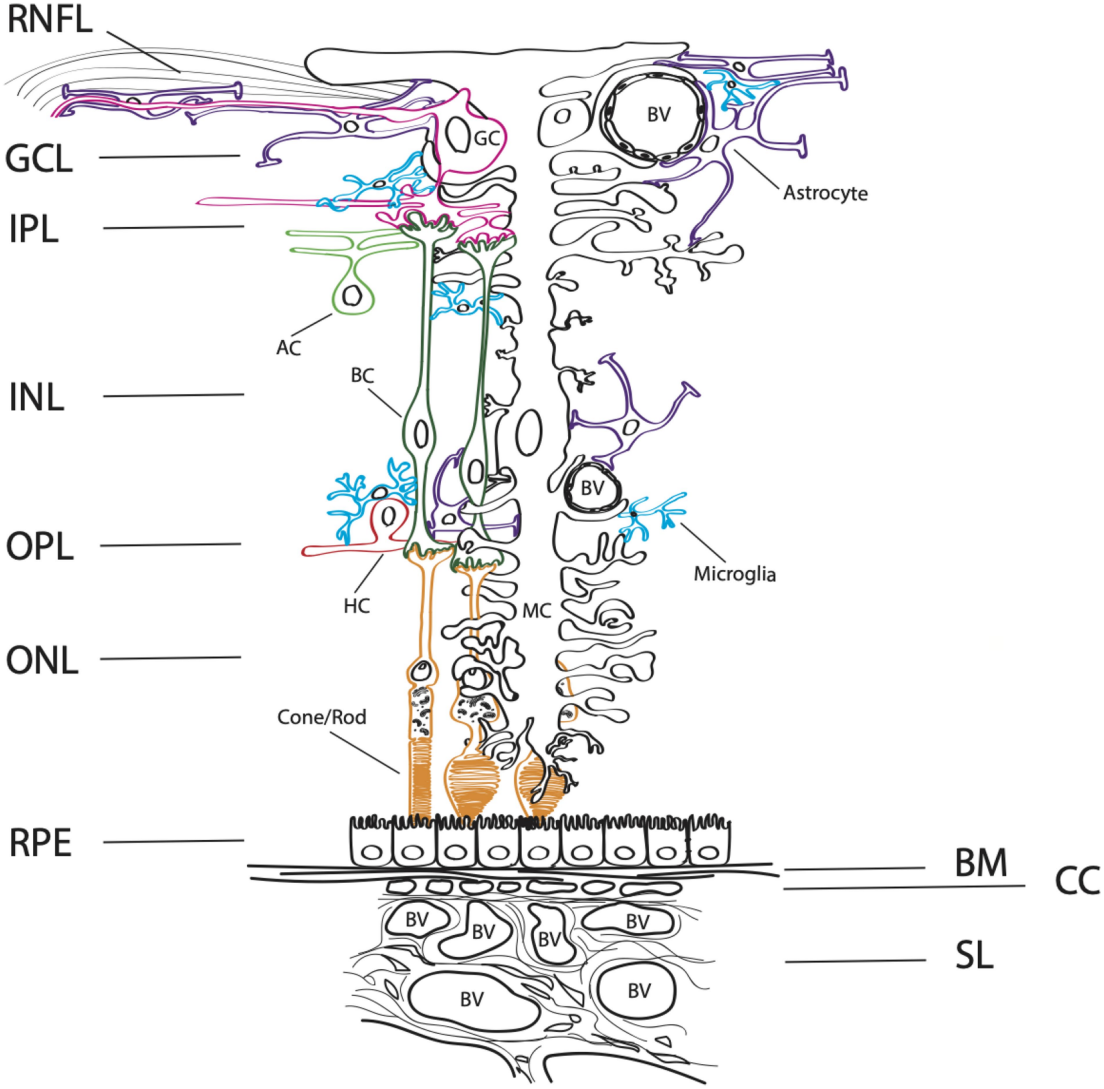
Complex architecture of the human retina. A schematic diagram of a cross-section through the retina. The layers of the neuroretina from inner to outer include: the retinal nerve fiber layer (RNFL), ganglion cell layer (GCL), inner plexiform layer (IPL), inner neuronal layer (INL), outer plexiform layer (OPL), and outer neuronal layer (ONL). Below the neuroretina is the retinal pigment epithelial layer (RPE) which is a monolayer of cells that sit on the extracellular matrix basement membrane, Bruch’s membrane (BM). The outer retinal is supplied by the choroidal blood circulatory system, comprised of the choriocapillaris (CC), the small vessel layer (SL) and the large vessel layer (not shown). Blood vessels (BV) are depicted within the figure. The retinal neurons in the inner retina include bipolar cells (BC, dark green), ganglion cells (GC, pink), amacrine cells (AC, light green), horizontal cells (HC, red), and rod and cone photoreceptors (orange). Microglia (blue), astrocytes (purple) and the Muller cells, a specialized astrocyte (MC, black) are supportive glial cell types that provide homeostasis and metabolic support for the retina.

**Figure 2:**
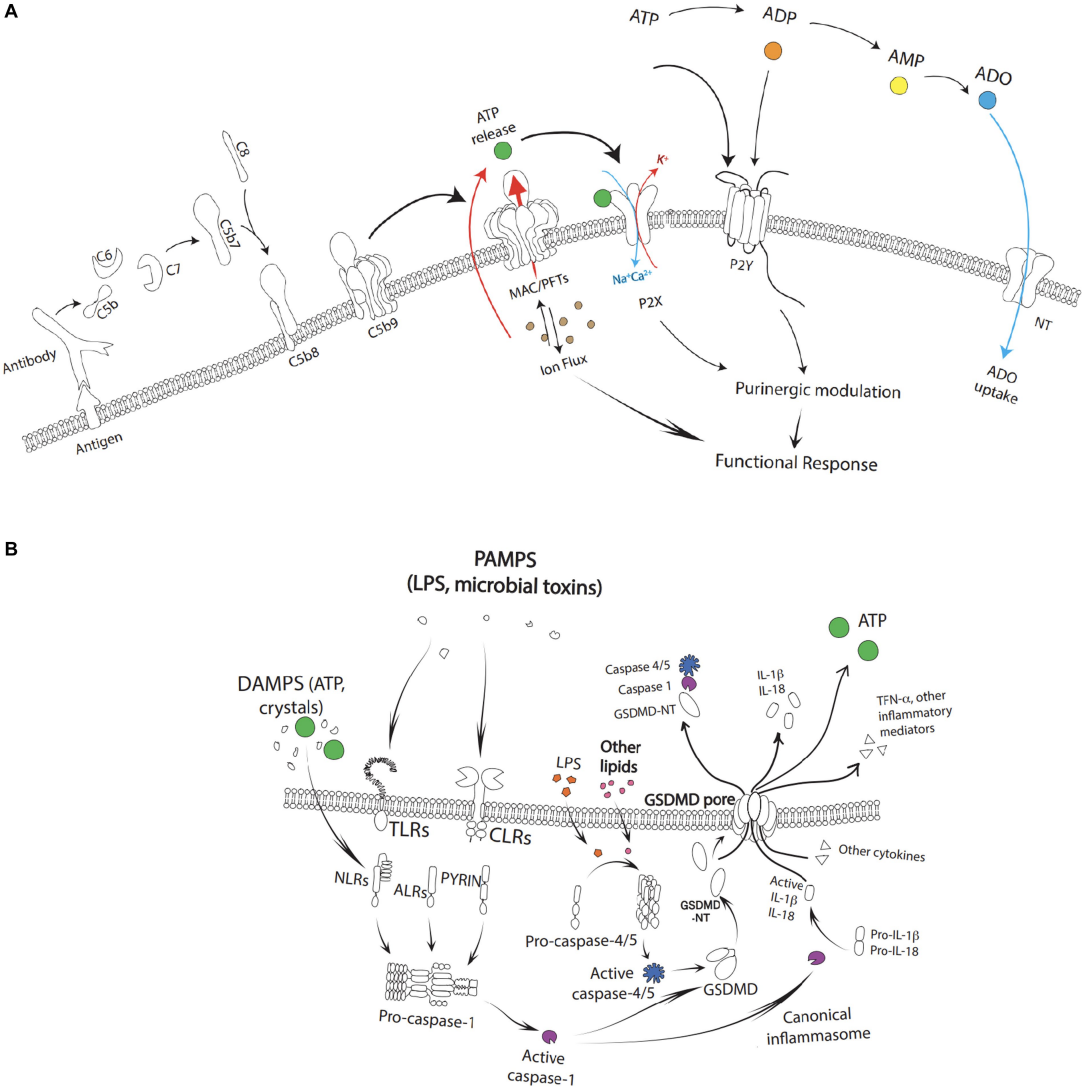
**(A)** Complement activation with the formation of the C5b-9(n) leading to the formation of a MAC/PFTs complex. The fully formed MAC creates large, 10 nm-wide pores initiating an ion flux cumulating in a calcium dependent cell lysis. As a result of cell lysis, high concentrations of ATP may be released into the local environment which can simulate P2X and activate downstream purinergic modification. Extracellular ATP similarly can activate P2Y. Extracellular adenosine is re-cycled back into the cytoplasm via the nucleoside transporters (NT). **(B)** Pathogen-associated molecular patterns (PAMPs) and damage-associated molecular patterns (DAMPs) such as toxins, pathogens, metabolites, crystalline substances, nucleic acids, ion flux, reactive oxygen species, and ATP stimulate toll-like receptors (TLRs) and c type lectin receptors (CLRs) leading to activation of NF-κB followed by the subsequent transcriptional upregulation of NOD-like receptor family pyrin domain containing 3 (NLRP3) and pro-interleukin-1β. The cascade as depicted leads to the formation of gasdermin-D (GSDMD) and pyroptotic cell death through activation of caspase-4/5, capase-1, and GSDMD-NT along with release of ATP and other pro-inflammatory factors such as IL-1b, IL-18, and TFN-α.

The organization of the retina has been well studied for its complex synaptic circuitry of retinal neurons, supported by two vascular beds, an inner (retinal) and outer (choroidal) retinal supply. The outer vascular bed is comprised of the choroidal circulation that supports the outer retina and plays an important homeostatic role for the retinal pigment epithelium (RPE) and photoreceptors (Figure 1). For cells of the inner neuroretina, RPE, choroid, and retinal vasculature, purinergic receptors from each receptor class are present (Yang et al., 2011; Wagner et al., 2013; Jacobson and Civan, 2016). This is important, as purinergic signaling has been implicated in the proliferation, survival, death, migration, and differentiation of retinal cells throughout development, aging, and in disease states. For a review on purinergic signaling in the inner retina, please see Ventura et al. (2019) and Sanderson et al. (2014). Here, we discuss the distribution and functions of purinergic receptors on cells present within the outer retina, including the RPE and choroid, that may influence and contribute to AMD pathogenesis (Figure 2).

### Retinal pigment epithelium

The RPE is considered a primary site of pathology in AMD (Strauss, 2005; Bhutto and Lutty, 2012). It is supported by Bruch’s membrane, an important extracellular matrix that separates the RPE from the choroidal blood supply. The RPE is positioned between the overlying outer segments of photoreceptors and the choroidal blood supply and combines the functions of epithelial and glial cells to act as both a barrier and supporting tissue for overlying photoreceptors (Strauss, 2005; Bhutto and Lutty, 2012). Indeed, communication between photoreceptors and the RPE is critical to retinal function and occurs through a small extracellular space that exists between the apical membrane of RPE cells and photoreceptors (Mitchell and Reigada, 2008). Located within this space is an abundance of enzymes and a highly structured extracellular matrix that allows for many functional interactions between the RPE and photoreceptors to take place (Mitchell and Reigada, 2008). For example, the RPE delivers nutrients from the choroidal blood supply to the photoreceptors, removes metabolic end products from photoreceptors, produces melanin granules to absorb stray light, and recycles molecules important for maintenance of the visual cycle (Strauss, 2005; Mitchell and Reigada, 2008; Bhutto and Lutty, 2012). The RPE also plays an integral role in the daily renewal of photoreceptors through the recycling and resynthesis of spent outer segments (Strauss, 2005; Mitchell and Reigada, 2008; Bhutto and Lutty, 2012).

While RPE cells have been found on exon level profiling to express all subtypes of purinergic receptors (Wagner et al., 2013), the distribution of P2X receptors in human RPE cells is not completely known and few studies have explored the role of P2X receptors within the outer retina. For example, Yang et al. (2011) found P2X7 receptor mRNA in human RPE cells and functional data indicating that in addition to P2X7, other P2X receptors such as P2X1, P2X2, P2X3, P2X4, and P2X5 may also be present. However, further studies are needed to understand the expression, localization, and functions of these receptors within RPE cells.

Perhaps the most well-characterized of these receptors in the RPE to date is the P2X7 receptor, which induces calcium signaling and the activation of numerous cellular pathways that lead to subsequent apoptosis in both native and cultured human RPE cells (Yang et al., 2011). RPE cell death also results in the release of proinflammatory cytokines and further unregulated release of ATP thereby increasing the vulnerability of other cells to ATP-induced apoptosis (Yang et al., 2011). Increasing concentrations of eATP released from stressed, injured, or damaged outer retinal cells (in the case of AMD and other retinal diseases) also influence aspects of their overall function as support cells for the inner neuroretina through cytokine and growth factor release and as a stimulant for proliferation and differentiation (Notomi et al., 2011; Niyadurupola et al., 2013; Notomi et al., 2013; Clapp et al., 2019; Platania et al., 2019).

An important aspect of P2X signaling in RPE cells is that under physiological circumstances, these cells can maintain homeostasis and prevent ATP-induced apoptosis by expressing high levels of the enzymes CD39 and CD73 within their membranes. CD39 is an ectonucleoside triphosphate diphosphohydrolase (NTPDase) that rapidly hydrolyzes ATP and ADP to AMP, while CD73 is an ectonucleotidase that degrades AMP to adenosine (Kukulski et al., 2011; Dwyer et al., 2020). Interestingly, after being exposed to inflammatory factors, Zhang et al. (2018) demonstrated that RPE cells can rapidly become CD73-negative. This was found to be the result of matrix metalloproteinase-9 (MMP-9)-mediated shedding of CD73 from the cell membrane of RPE after exposure to inflammatory factors *in vitro*, leading to impaired immune suppression, increased concentrations of eATP, and accelerated local inflammation in the AMD environment (Zhang et al., 2018).

### Immune cells

All immune cells, whether of the myeloid or lymphoid lineage, express at least one P2X receptor subtype, and many express all seven subtypes (Burnstock, 2016). Within the outer retina and choroid, these immune cells include resident retinal microglia, mast cells, lymphocytes, monocytes, and dendritic cells (Sarma and Ward, 2011; Merle et al., 2015a,b; Di Virgilio et al., 2017; Behnke et al., 2020; Di Virgilio et al., 2020; Ogura et al., 2020).

### Microglia

Microglia are resident professional phagocytes of the CNS, similar in function to blood-borne peripheral immune cells including monocytes, macrophages, and lymphocytes. They possess a high density of P2X7 receptors (Illes et al., 2017, 2020) in addition to P2X1 and P2X4 (Di Virgilio et al., 2020). Further, their ability to function as scavengers by migrating toward and clearing insoluble photo-oxidized material found in drusen has been shown to result from purinergic signaling interactions, such as in the case of ATP and P2X7 receptor activation (Gu and Wiley, 2018). Indeed, the heterogeneous expression of P2X1, P2X4, and P2X7 receptors on the surface of macrophages with varying ATP affinities may enable fine-tuning of macrophage responses to ATP (Guo et al., 2007; Adinolfi et al., 2018). This can result in distinct desensitization kinetics and diverse intracellular transduction pathways that contribute to numerous pro-inflammatory pathways in a concentration-dependent manner (Adinolfi et al., 2018).

Genetic association studies have also investigated this link by identifying a unique haplotype containing a heterotrimeric combination of P2X4 and P2X7 subunits that increases an individual’s risk of developing AMD due to impaired P2X7 function. This results in reduced phagocytic capacity of macrophages, delayed clearance of apoptotic cells, and leakage of ATP from necrotic cells (Gu et al., 2013). Furthermore, P2X7-null mice models demonstrated reduced blood-borne macrophage phagocytosis activity resulting in thickening of Bruch’s membrane, RPE dysfunction, and retinal stress at 12 months of age (Vessey et al., 2017), followed by Bruch’s membrane thickening, RPE cell loss, retinal functional deficits, and signs of inflammation between the RPE and photoreceptors at 18 months of age – phenotypic characteristics consistent with early AMD (Vessey et al., 2017). Taken together, communication between macrophages occurs, in part, via purinergic signaling.

Other studies also support the role of ATP in regulating macrophage chemotaxis and macrophage activation (Kronlage et al., 2010; Junger, 2011; Sakaki et al., 2013). This outlines a potential mechanism for the role of purinergic signaling in the pathogenesis of AMD, whereby immune cells present within the outer retina are no longer able to manage the task of removing the constant supply of photoreceptor debris, leading to the progressive and damaging accumulation of drusen within Bruch’s membrane, activation of bystander cells, worsening nutrient and oxygen support for the RPE, and a vicious cycle of RPE failure, neuronal cell death, and central vision loss (Strauss, 2005; Zumerle et al., 2019).

### Mast cells

Mast cells (MCs) are recognized as key components of inflammatory reactions and are implicated in several inflammatory diseases. They are responsive to toxins and microbes, as well as substances such as advanced glycation end products, complement factors, C-reactive protein, and ATP, all of which are implicated in AMD (Ogura et al., 2020). Of the seven P2X receptors, only five (P2X1, P2X3, P2X4, P2X6, and P2X7) are expressed by MCs (Wareham et al., 2009; Wareham and Seward, 2016). These receptors play an important role in regulating MC activities, such as calcium influx and degranulation that results in the release of many pre-stored inflammatory mediators (Bulanova and Bulfone-Paus, 2010). Inflammatory mediators include IL-1β, Nuclear factor kappa B (NF-κB), tumor necrosis factor α (TNF-α), serotonin, and kinins, along with the synthesis and secretion of an array of cytokines, chemokines, prostaglandins, leukotrienes, and growth and angiogenesis factors (i.e., platelet-derived growth factor and VEGF) (Galli and Tsai, 2008; Bulanova and Bulfone-Paus, 2010; Kurashima et al., 2012; Theoharides et al., 2012; Shieh et al., 2014; Salcman et al., 2021). These MC components can modulate the activity of cells in their proximity and lead to the generation of reactive oxygen species (ROS), promotion of chemotaxis, altered phagocytosis, degradation of underlying extracellular matrix (ECM), and other events contributing to an overall increase in inflammation (Bhutto et al., 2016; Kempuraj et al., 2016; Caraffa et al., 2018).

In the pathogenesis of AMD, MC-derived tryptase release also results in the breakdown of collagens and activation of MMPs that degrade choroidal stroma and Bruch’s membrane. This leads to thinning of the choroid and degeneration of the RPE, both of which are hallmarks of GA (Ogura et al., 2020). Additionally, MC activation has been implicated in choroidal neovascularization through granzyme B release through intracellular immune-mediated cell death and extracellular ECM degradation (Matsubara et al., 2020). This results in remodeling of the ECM in Bruch’s membrane, breakdown of the blood-retina barrier, and slowing of metabolite transport between the choroidal blood supply and retina, which can contribute to drusen deposition, vascular leakage, disruption of choroidal endothelial cell function, and the release of sequestered VEGF from Bruch’s membrane (Matsubara et al., 2020).

### Choroid and retinal vasculature

The retina is nourished by two independent vascular supplies (Figure 1). The outer retina and photoreceptors are fed by the choroidal vasculature that lies directly beneath the photoreceptors and the RPE, while the inner retina is served by intrinsic retinal vasculature, branches of the central retinal artery that enter at the optic disc (Newman, 2015). As autonomic innervation is absent in the generation of retinal vascular tone, the tone of these vessels must be generated by intrinsic mechanisms such as the release of vasoactive agents from neurons, glial cells, and vascular endothelial cells (Kur et al., 2012; Newman, 2015). For this reason, purinergic signaling involving ATP has been explored as a mechanism to generate tone in retinal arterioles (Kur and Newman, 2014). Indeed, experiments have demonstrated that a reduction of endogenous eATP levels leads to arteriole dilation, while an increase in eATP levels leads to vessel constriction through altered P2X1 receptor activity (Kur et al., 2012; Newman, 2015).

Purinergic signaling also results in choroidal and retinal neovascularization through remodeling of existing vasculature and proteolytic degradation of the endothelial basal membrane and surrounding extracellular matrix via MMP-2 and MMP-9 activation (Yancopoulos et al., 2000; Berglin et al., 2003). Additionally, stimulation of P2X receptors promotes VEGF release and alters endothelial barrier properties depending on the type of receptors present and the local concentration of the nucleotides within the vasculature (Adinolfi et al., 2018). For instance, chronic P2X receptor activation with ATP acting as a danger-associated molecular pattern (DAMP) at high concentrations leads to endothelial barrier destabilization and edema formation through impaired Müller cell function in the induction, maintenance, and proper functioning of the blood–retinal barrier (Shen et al., 2012; Wakx et al., 2016). Together, these processes can contribute to retinal degeneration under pathologic conditions such as the proinflammatory environment seen in AMD.

## Mechanisms of ATP release

Broadly, mechanisms of intermittent ATP release can be the result of (1) cell damage or cell death (e.g., complement activation and MAC deposition, osmotic swelling, ischemia, inflammation, or apoptosis leading to the passive leakage of ATP from cells), (2) vesicular release, or (3) channel-mediated release (Bulanova and Bulfone-Paus, 2010). However, sustained ATP release which is likely the ATP release of pathophysiological significance can also result from a multiplicity of pathways (Lazarowski et al., 2003; Dale et al., 2023; Di Virgilio et al., 2023; Shinozaki et al., 2023). Within the outer retina, several processes that contribute to AMD pathogenesis lead to ATP release (Figure 2). For example, activation of the complement cascade results in ATP release from MAC deposition leading to inflammasome activation, the release of pore-forming gasdermins, and pyroptosis. ATP release can also act as a feedforward method to trigger P2X receptors and further promote cell degeneration in the AMD outer retina. Recently, apoptosis has also been shown to release ATP as a “find me” signal through Pannexin 1 channels (Medina et al., 2020).

### Complement system

The complement system plays a central role in AMD pathogenesis, along with aspects of cellular immunity and homeostasis. It consists of a network of proteins that can be sequentially cleaved and activated through any of three distinct pathways: the classical pathway, the lectin pathway, and/or the alternative pathway (Ricklin et al., 2010; Sarma and Ward, 2011; Merle et al., 2015a,b). Each of these pathways converge in the terminal pathway of the complement system, which results in the formation of the C5b-9(n) MAC complex. Fully formed MAC creates large, 10 nm-wide, pores in the membranes of pathogens and vulnerable host cells and can result in calcium dependent cell lysis (Sarma and Ward, 2011; Merle et al., 2015a,b). For a comprehensive review of complement and its role in AMD, please see Armento et al. (2021).

As a result of cell lysis, high concentrations of ATP may be released into the local environment. This can stimulate P2X receptors and influence the recruitment and activation of numerous inflammatory cells such as retinal microglia, mast cells, and circulating lymphocytes, monocytes, and macrophages, as described above (Ricklin et al., 2010; Sarma and Ward, 2011; Merle et al., 2015a,b; Behnke et al., 2020; Ogura et al., 2020). Ultimately, this amplification loop can: (1) induce changes in the composition of Bruch’s membrane, the choriocapillaris, and ECM, (2) impair transport properties, alter lipid metabolism, and result in the accumulation of drusen, and (3) lead to chronic inflammation, oxidative stress, and altered energy metabolism as seen in the pathogenesis of AMD (Armento et al., 2021).

These findings are echoed by genetic studies where over 33 different loci associated with aspects of the complement system, ECM remodeling, and other pathways such as cholesterol metabolism have demonstrated an increased risk for the development of AMD (Klein et al., 2005; Schramm et al., 2014; Black and Clark, 2016). Complement activation can also alter the expression of MMP-2 and MMP-9 in various cell types, including RPE (Bandyopadhyay and Rohrer, 2012). As discussed above, this can result in ECM turnover, neovascularization due to imbalances in VEGF secretion, altered ATP metabolism due to interactions with extracellular nucleosides, and increased purinergic signaling.

### Sublytic MAC formation and pore forming toxins

MAC deposition does not always result in lysis of host cells due to the presence of regulatory proteins and active repair processes. For example, active repair processes such as MAC plugging, exocytosis, and endocytosis repair cell membranes and remove MAC pores before lysis can take place to limit sustained elevations in intracellular calcium (Kunchithapautham and Rohrer, 2011). Other regulatory processes include CD59, a membrane-bound GPI-anchored protein that inhibits the addition of C9 into the C5b-8/9 complex on host cells, which limits mean MAC lesion size (Kunchithapautham and Rohrer, 2011). Soluble inhibitors such as vitronectin or clusterin that bind to the C5b-7 structure of the MAC can also prevent its attachment to cell membranes, rendering it water-soluble and inactive (Kunchithapautham and Rohrer, 2011). Notably, these changes in MAC lesion size and binding affect the kinetics of ATP release and ion flux thereby influencing aspects of purinergic signaling.

Under sublytic conditions, several effects have been described that are hypothesized to contribute to the development and progression of both dry and wet forms of AMD. For instance, sublytic MAC formation can activate signaling pathways related to calcium, receptor tyrosine kinases, phospholipase C, protein kinase C, phospholipase 2α, and other extracellular signal-regulated kinases (Cybulsky et al., 2005; Fosbrink et al., 2005). This can lead to changes in cellular response including secretion, adherence, aggregation, chemotaxis, cell division, and impacts on membrane function (Bohana-Kashtan et al., 2004). In RPE cells, sublytic MAC increases the production of cytokines IL-6, IL-8, and MCP-1, which may contribute to early AMD (Lueck et al., 2011). Increased expression of MMP-2 and MMP-9 and VEGF are also associated with sublytic MAC formation on RPE and correlate with both remodeling of the choriocapillaris and neovascular processes seen in wet AMD (Thurman et al., 2009; Lueck et al., 2011). This is because VEGF, present in granular vesicles, is secreted via exocytosis following depolarization of cell membranes through activation of voltage-gated calcium channels. Calcium influx also activates the Ras/Erk pathway known to be involved in the regulated secretion of VEGF (Kunchithapautham and Rohrer, 2011). Additionally, P2X7 receptor activation also triggers VEGF release (Hill et al., 2010; Adinolfi et al., 2012). Thus, sublytic MAC formation and purinergic signaling influences intracellular signaling pathways that result in growth factor secretion (Lueck et al., 2011).

Various membrane pore-forming toxins, such as α-haemolysin, leukotoxin, and α-toxin, have also been shown to exert their toxic effects through autocrine and paracrine signaling in human erythrocytes (Birke et al., 2013) leading to complement-mediated lysis amplified by ATP release and P2X receptor activation (Birke et al., 2013). Additionally, amyloid-β protein aggregates and other pore forming toxins may lead to sublytic membrane damage and subsequent release of cellular components such as ATP, IL-1β, and IL-18 (Sanz et al., 2009; Ciudad et al., 2020). However, the underlying mechanisms and processes surrounding complement amplification, MAC deposition, and P2X receptor activation are not yet fully understood. We hypothesize that MAC deposition leads to an increase in eATP and subsequent P2X receptor activation. This results in an enhancement of ion flux, which has an impact on mitochondrial potential, the formation of ROS, inflammasome activation, and other intracellular changes leading to a feedback loop that allows for more MAC deposition. Further research is needed to establish these connections.

Overall, sublytic MAC and pore formation results in the remodeling of the choriocapillaris which contributes to the buildup of drusen, enhances complement activation and NLRP3 inflammasome activity, and leads to increased inflammation through cytokine release and recruitment of immune cells. Chronic inflammatory changes impact the overlying RPE, and the outer retina responds through additional signaling resulting in CNV, or regression of the choriocapillaris forming “ghost” vessels, subsequent RPE loss, and photoreceptor death in GA (Kumar-Singh, 2019).

### NLRP3 inflammasome activation

Inflammasomes are multimolecular complexes comprised of three protein constituents: a NOD-like receptor, the adaptor protein apoptosis-associated speck-like protein containing a caspase recruitment domain (ASC), and pro-caspase 1 (Latz et al., 2013). Their activation consists of a two-step process in which both an initial priming signal and an activating signal are required (Latz et al., 2013). The initial priming signal is initiated by pathogen-associated molecular patterns (PAMPs) that stimulate toll-like receptors (TLRs) leading to the activation of NF-κB followed by the subsequent transcriptional upregulation of NOD-like receptor family pyrin domain containing 3 (NLRP3) and pro-interleukin-1β (Latz et al., 2013; Kelley et al., 2019). This is especially important in non-immune cells such as the RPE where basal expression levels are considered insufficient to initiate inflammasome assembly (Tseng et al., 2013; Narendran et al., 2021). Next, an activation signal is provided by a broad variety of molecules classified as either PAMPs or damage-associated molecular patterns (DAMPs) such as toxins, pathogens, metabolites, crystalline substances, nucleic acids, ion flux, reactive oxygen species, and ATP (Latz et al., 2013; Malik and Kanneganti, 2017; Zheng et al., 2020). In the case of P2X7 activation, eATP acting as a DAMP is detected by P2X7. Following activation, inflammasomes lead to a unique inflammatory programmed cell death pathway known as pyroptosis (Kelley et al., 2019).

Pyroptosis is executed by a family of pore-forming proteins known as gasdermins (GSDMs) (Shi et al., 2015). In humans, the current members of the GSDM family include GSDMA, GSDMB, GSDMC, GSDMD, and GSDME, which contain an autoinhibitory carboxyterminal domain and a pore-forming amino-terminal domain responsible for perforating the plasma membrane of cells (Orning et al., 2019; Broz et al., 2020; Liu et al., 2021). Typically, pyroptotic cell death initiates following the activation of the NLRP3 inflammasome and most often results in GSDMD pore formation and release of ATP (He et al., 2015; Kayagaki et al., 2015; Shi et al., 2015). Proinflammatory cytokines such as IL-1β and IL-18 are also released through the nonselective 10–14 nM gasdermin pore (Latz et al., 2013; Yu et al., 2021) which induce both inflammatory an apoptotic effects (Martinon et al., 2002; Ambati et al., 2013). However, while there are cytotoxic effects of IL-18 and IL-1β on the RPE, studies have also shown beneficial effects of inflammasome-mediated IL-18 release through the inhibition of neovascularization in an acute laser-induced injury model of neovascular AMD (Doyle et al., 2012). These contrasting findings imply that a single factor (IL-18) or pathway (NLRP3 inflammasome activation) can be simultaneously anti-angiogenic and destructive to the RPE and that Toll-like receptor 3 (TLR3) activation may be beneficial in terms of decreasing choroidal neovascularization while also promoting RPE degeneration (Ambati et al., 2013). In contrast to the reported anti-angiogenic effects of IL-18, IL-1β promotes neovascularization (Lavalette et al., 2011).

Based on the mechanisms described above, activation of the NLRP3 inflammasome and various gasdermin proteins have been implicated in the pathogenesis of AMD and several pathways have been suggested to trigger inflammasome activation in the outer retina including lipofuscin component A2E, accumulated Alu RNA, drusen components, amyloid-β, lipid peroxidation products, photooxidative damage, lysosomal destabilizations, particulate matter, overexpression of VEGF, and eATP (Ambati et al., 2013; Kerur et al., 2013). For example, the formation of amyloid-β oligomers (AβOs), which are aggregates of amyloid-β peptides and a major proinflammatory component of drusen (Luibl et al., 2006), can lead to RPE degeneration and GA through AβO-induced priming, assembly, and activation of the NLRP3 inflammasome in RPE cells. This occurs through a P2X7-mediated pathway, in which amyloid-β protein aggregates form a conductivity pore resulting in membrane damage and subsequent release of cellular components such as ATP and inflammatory mediators (Sanz et al., 2009; Ciudad et al., 2020). Like other mechanisms of NLRP3 inflammasome activation, AβO-induced AMD models have been demonstrated to result in the expression of GSDMD (Sun et al., 2018), along with RPE cytotoxicity driven by mitochondrial dysfunction and ROS formation (Sorbara and Girardin, 2011; Zhou et al., 2011).

Additionally, repetitive element-derived *Alu* RNA transcripts, non-canonical targets of DICER1-mediated enzymatic degradation, accumulate in human GA following the loss of DICER1 expression and are capable of activating P2X7 and the NLRP3 inflammasome to cause cell death of the retinal pigment epithelium in GA (Fowler et al., 2014). This is because *Alu* RNA transcripts can function as both priming and activating signals for inflammasome signaling (Ambati et al., 2013; Kerur et al., 2013). As a result of this pathway and the fact that *Alu* RNA transcripts require reverse transcriptase, multiple nucleoside reverse transcriptase inhibitors (NRTIs) have been investigated and found to be efficacious in inhibiting P2X7-mediated NLRP3 inflammasome activation in mouse models of GA, CNV, and other P2X7 driven diseases (Fowler et al., 2014).

Links between purinergic signaling and NLRP3 inflammasome activation are also well-defined as a result of ATP acting as a paracrine or autocrine signal in response to cell death or other stimuli (i.e., increased pressure, hypoxic injury, or complement-mediated damage). In these scenarios, the high amount of passive ATP release from cells activates the inflammasome through a P2X7R-dependent pyroptotic cell death pathway (Yang et al., 2015). Activated caspase-11 may also cleave pannexin-1 channels, inducing ATP release and P2X7R-related pyroptosis (Yang et al., 2015). Other nucleotide metabolites such as ADP, UTP, UDP, UDP glucose, and adenosine, along with other members of the purinergic receptor family (i.e., P2X, P2Y, and P1 receptors), may also contribute through complex purinergic signaling networks (Gombault et al., 2013).

Taken together, an increasing body of evidence suggests that the retina can respond to diverse danger signals including unregulated ATP release via purinergic signaling leading to NLRP3 inflammasome activation (Gombault et al., 2013; Gao et al., 2015; Yang et al., 2020), GSDM pore formation, and pyroptosis. Therefore, inhibition of P2X receptors and NLRP3 activation has been identified as putative drug targets in several models of AMD progression by delaying RPE degeneration in GA and/or slowing RPE barrier breakdown and neovascularization in CNV.

## Conclusion

**Figure 3:**
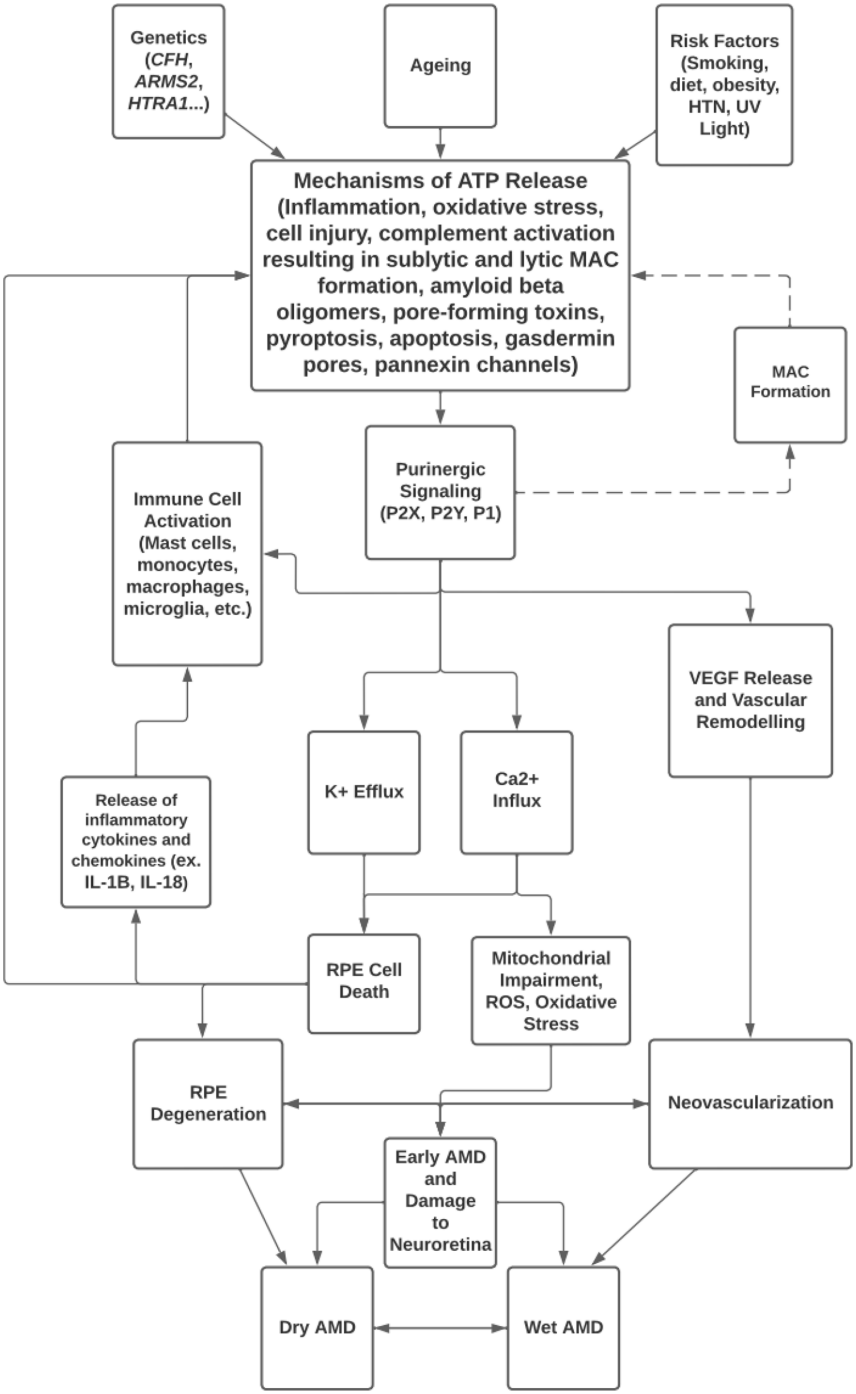
Overview of mechanisms of ATP release and purinergic signaling in the pathogenesis of AMD.

Purinergic signaling has been investigated for its role in the development of ocular pathologies such as AMD, glaucoma, and diabetic retinopathy. Despite the emergence of anti-VEGF agents to treat the wet form of late AMD, and the recently FDA-approved pegcetacoplan, a complement C3 inhibitor, to slow the progression of the dry form of late AMD, there are no approved drugs available to prevent the development of wet or dry AMD. For this reason, potential crosstalk between known contributors to AMD, such as complement dysregulation and inflammasome activation, and other cellular systems, such as purinergic signaling, must be considered (Figure 3).

As outlined in this review, the current literature surrounding purinergic signaling and AMD pathogenesis has focused primarily on the role of P2X7 receptor signaling. However, P2X7 has the highest EC50 for ATP in the P2X receptor family and may not be physiologically relevant throughout all stages of the development and progression of AMD, especially in the early stages of the disease. On the other hand, in advanced stages and under certain conditions, mechanisms may allow for P2X7 receptor activation through altered regulatory proteins, repair processes, and interactions between mediators such as MMPs and ectonucleosides leading to increased concentrations of ATP, increased purinergic signaling, and accelerated local inflammation contributing to the AMD pathogenesis.

To fully understand the potential roles that purinergic signaling plays in AMD, more research is needed surrounding the expression, distribution, functions, and interactions of P2X receptors with other systems, such as complement activation, within cells of the outer retina, RPE, choroid, retinal vasculature, and the immune system. This must include further characterization of both homotrimeric purinergic receptors, such as P2X1, P2X2, P2X3, P2X4, P2X5, and P2X6, along with heterotrimeric receptors that can exhibit novel properties and functions.

Finally, while there are numerous mechanisms for ATP release within the outer retina, the role of purinergic signaling in both lytic and sublytic processes should be explored in the context of how these processes may amplify complement-induced lysis, a mechanism that has not yet been fully elucidated. This may involve processes that make cells more vulnerable to MAC deposition following P2X activation, such as crosstalk between complement and P2X receptor signaling, MMP-9 activation, and other spatial and temporal aspects of ATP release. In determining how these processes can influence and be influenced by purinergic signaling, it will improve our understanding of the mechanisms that drive AMD pathogenesis, which is critical in developing treatment strategies that prevent or slow the progression of the disease.

## Author contributions

HM performed the data collection and analysis. HM, KJ, and JM wrote the manuscript. JM and CC conceived the review, obtained funding, and critically revised the manuscript. All authors have read and approved the final manuscript.

## Conflict of interest

CC was employed by Paragon Ventures Inc.

The remaining authors declare that the research was conducted in the absence of any commercial or financial relationships that could be construed as a potential conflict of interest.

## Publisher’s note

All claims expressed in this article are solely those of the authors and do not necessarily represent those of their affiliated organizations, or those of the publisher, the editors and the reviewers. Any product that may be evaluated in this article, or claim that may be made by its manufacturer, is not guaranteed or endorsed by the publisher.
